# Are Platelets Cells? And if Yes, are They Immune Cells?

**DOI:** 10.3389/fimmu.2015.00070

**Published:** 2015-02-20

**Authors:** Olivier Garraud, Fabrice Cognasse

**Affiliations:** ^1^Institut National de la Transfusion Sanguine, Paris, France; ^2^EA3064, Université de Lyon, Saint-Etienne, France; ^3^Etablissement Français du Sang Auvergne-Loire, Saint-Etienne, France

**Keywords:** platelets, hemostasis, cell physiology, cell biology, immunophysiology, immunopathology, cytokines

## Abstract

Small fragments circulating in the blood were formally identified by the end of the nineteenth century, and it was suggested that they assisted coagulation via interactions with vessel endothelia. Wright, at the beginning of the twentieth century, identified their bone-marrow origin. For long, platelets have been considered sticky assistants of hemostasis and pollutants of blood or tissue samples; they were just cell fragments. As such, however, they were acknowledged as immunizing (to specific HPA and HLA markers): the platelet’s dark face. The enlightened face showed that besides hemostasis, platelets contained factors involved in healing. As early as 1930s, platelets entered the arsenal of medicines were transfused, and were soon manipulated to become a kind of glue to repair damaged tissues. Some gladly categorized platelets as cells but they were certainly not fully licensed as such for cell physiologists. Actually, platelets possess almost every characteristic of cells, apart from being capable of organizing their genes: they have neither a nucleus nor genes. This view prevailed until it became evident that platelets play a role in homeostasis and interact with cells other than with vascular endothelial cells; then began the era of physiological and also pathological inflammation. Platelets have now entered the field of immunity as inflammatory cells. Does assistance to immune cells itself suffice to license a cell as an “immune cell”? Platelets prove capable of sensing different types of signals and organizing an appropriate response. Many cells can do that. However, platelets can use a complete signalosome (apart from the last transcription step, though it is likely that this step can be circumvented by retrotranscribing RNA messages). The question has also arisen as to whether platelets can present antigen via their abundantly expressed MHC class I molecules. In combination, these properties argue in favor of allowing platelets the title of immune cells.

## Introduction

It has become usual in the medicine and physiology community to present platelets as “cells” that are indispensable to primary hemostasis (their function in thrombosis is sometimes ignored); but still today, it is not uncommon that representatives of the pathologist community refute use of the word “cells” for platelets. Platelets, for many, are dust or pollutants at worse, and cell debris at best, though no one doubts their hemostatic functions. Two distinct arguments may be considered in an attempt to explain their reluctance to recognize platelets as cells:

1. *Ex vivo*, platelets usually “contaminate” histological preparations; because they have no nucleus and they apparently are “solely” cytoplasm fractions, and platelets differ from cells forming tissues and also from the regular blood cells: they resemble impurities.
2. *In vivo*, platelet “puree,” and not mandatorily fresh, alive, platelets within platelet components (PCs) for transfusion purposes, can also achieve hemostasis in emergency conditions and stop bleeding. This is how a large proportion of thawed PCs [a type of blood component (BC) rarely used] behaves.

## Discovery of Platelets and Their Functions

The initial discovery of platelets is disputed. Was Boyle (end of the seventeenth century), Donné (early nineteenth century), or Bizzozzero (end of the nineteenth century) the real discoverer? It is generally considered that the acknowledgment there is a third cellular element in blood besides, and independent of, erythrocytes and leukocytes, is attributed to Bizzozzero, around 1881–1882. This Italian (Lombardy) physician and researcher also very elegantly acknowledged the role of platelets not only in hemostasis but also in thrombosis. Prior to Bizzozzero, and indeed as early as the seventeenth century, platelets had been suspected. For instance, van Leewenhoek, the Dutch microscopist who delivered amazing, seminal observations of natural life, reported precise observations of platelets around 1675. Hewson (around 1780) reported undefined blood particles. At that time, platelets bore names such as particles, corpuscles, and globules. Important information on their size, form change, granular content, and ability to emit filaments was thus available quite early. However, at this time, platelets were often considered to be of leukocyte origin and to be degenerate or degraded. Some observers favored an erythrocyte origin (such as erythrocyte precursors or haemoblasts). Bizzozzero set up experiments to see platelets in veins, and also as circulating in the mesentery of living animals. He found that they were unrelated to erythrocytes and leukocytes, showed their role in hemostasis and thrombosis, and predicted the intimate relationship of platelets and leukocytes in the latter (and in other functions), as previously only leukocytes were visible in clots. The “only” discovery that Bizzozzero did not make is the acknowledgment of the bone marrow megakaryocyte ancestry of platelets, which Wright did in 1910 (1, 2).

## Why are Platelets Sometimes Not Recognized as Cells?

Platelets are non-nucleated cell elements that, clearly, result from fractionation of bone marrow megakaryocytes (MKs). During differentiation, MKs are exposed to constantly increasing pH and pO_2_ until reaching the sinuses, where platelets are released from proplatelets supported by shear from the blood flow. Expression of CD34 decreases as MKs mature, while expression of CD41 and CD42b increases (3). In the final step of MK development, platelets are released, and MK cytoskeletal reorganization is an important intracellular process for these morphologic changes. The correlation between cytoskeleton reorganization and proplatelet formation has not been completely clarified. Serotonin is thought to modulate cell migration and remodeling through activation of cytoskeleton reorganization, depending on the Rho/ROCK and Erk1/2 pathways, and 5HT_1A/1B/1D_ and 5HT4 receptors (4).

As such, platelets contain (inherit) MK cytoplasm complete with granules, mitochondria, and mRNA. Indeed, anucleate platelets lack genomic DNA but inherit a diverse array of functional coding or non-coding RNAs and translational machinery from their parent cells, enabling activated platelets to synthesize proteins, which suggests the possibility of post transcriptional gene regulation in platelets (5–8). The soluble proteins stored in the α-granule matrix, such as von Willebrand factor and thrombospondin, are derived via exclusive synthesis in the MKs, or for proteins such as fibrinogen and albumin, through endocytosis of plasma proteins (9).

Thus, platelets resemble cell fragments rather than fully licensed cells. One of the strongest arguments is probably that platelets have no genes to reorganize, because they have no nucleus and supporting DNA material (apart from the mitochondrial genome).

What about comparison with erythrocytes? Erythrocytes are not denied the qualification of cells though they have neither a nucleus nor genes to reorganize. However, the non-nucleated status of erythrocytes only pertains to mammals, not birds or reptiles. Erythrocytes in mammals evolved from erythroblasts, which are nucleated, and the process of enucleation is finite: one nucleated erythroblast gives rise to one erythrocyte with no intermediate division or transformation (10, 11). MKs, when forced to terminal maturation, separate into a degraded nucleus and platelets, emphasizing the nature of platelets, which are “only” cytoplasm fragments. An MK may produce 10–20 proplatelets, each of which starts as a blunt protrusion that over time elongates, thins, and branches repeatedly. The proplatelets extend into sinusoidal spaces, where they detach and fragment into individual platelets, giving rise to about 2000–5000 new platelets. Each day, in every human, approximately 1 × 10^11^ platelets are produced by the cytoplasmic fragmentation of MKs (12–15).

Although it is well established that platelets originate from MKs, the mechanisms by which they are formed and released remains controversial. Three models of platelet formation have been proposed: (1) cytoplasmic fragmentation, (2) platelet budding, and (3) proplatelet formation (13, 16, 17).

One must note that this classical picture of thrombopoiesis is unique (if one accepts the long-debated possibility that there is extramedullary thrombopoiesis, possibly in the lung). It has, however, recently been shown that there is a modest but definite thrombopoiesis in the circulation, as platelets give rise themselves to buds and extrusions distinct from platelet micro-particles (PMPs), turning into pro- and then pre-platelets (5–8, 18, 19). This is not a characteristic of a cell fragment without autonomy. This is, if further evidence is needed, a very strong argument in favor of platelets’ classification as cells. Platelets also form the link between thrombosis and inflammation through the production of microparticles (MPs). PMPs are phospholipid vesicles (100–1000 nm) released after budding from the platelet plasma membrane. Platelets shed these membrane vesicles after stimulation with physiological agonists such as thrombin or collagen, in response to high shear stress (e.g., in severe stenosis) or in the presence of danger signals. As a result, PMP express the same antigens as their parent cells, i.e., GPIIb–IIIa, GPIb, CD31, CD61, and CD62P. This distinguishes them from MPs derived from other cell types (red blood cells, leukocytes, monocytes, endothelial cells). PMPs thus make up between 70 and 90% of the circulating vesicles. PMPs differ from exosomes by their size, and also due to the fact that they are not derived from exocytosis of multivesicular bodies (20–24).

## Platelets Behave as Cells

Platelets share some very important properties of cells. We list nine here – in physiological order of appearance – but there are many more.

1. Platelets display receptors for a variety of moieties, collectively termed “stimuli,” but individually are quite different in nature. Platelets have glycoproteins (GPs) that sense exposed vascular sub-endothelium structures after vessel stress, insult, or attrition (including mechanical erosion and aging). Platelets can sense non-self infectious danger signals via a panoply of receptors detailed in a companion article. Platelets also display alarmins that can sense self-injury (25–27).
2. Platelets can respond to soluble molecules, via their receptors, and this can be particularly evidenced for thrombin or thrombin-derived peptides; hence, platelets are reactive to agonists and also to antagonists of such hemostatic/thrombotic factors. Additionally, platelets can react to biological response modifiers (BRMs) such as cytokines and chemokines, and become activated or inhibited. Some new drugs and biologics exploit those discerning properties (28–36).
3. Platelets comprise a complete and functional signalosome. Upon stimulation, they can phosphorylate a cascade of signaling molecules upstream of NFkB. NFkB is a crucial molecule in platelet physiology (37–42).
4. The platelet proteome is currently being investigated to determine the effects of treatments inflicted on platelets, such as for PC processing prior to transfusion. It is quite impressive, with more than 1000 proteins currently identified, and more being discovered regularly; 300–350 of those proteins have been proven as secreted by platelets (43). Platelet originating proteins come from three origins: (i) they may be inherited from the MK, (ii) they are absorbed from neighboring fluids and especially plasma [platelets have been described as “sponges” (44)], and (iii) they can be produced *de novo*, using a retrotranscription RNA process and spliceosome. This latter property further justifies the qualification of “cell” for platelets (45–51).
5. Platelets are docked with proteins/GPs for several, and distinct, purposes, including hemostasis, thrombosis, sensing, natural anti-infection (bacterial, viral, perhaps fungal) defense, chemo-attraction, cell communication, angiogenesis, healing, and tissue repair. It is clear, though under-evaluated in terms of physiological value, that platelets make distinction among the “dangers” they face. They can decipher vascular insult and presence of (circulating) infectious pathogens or components of the microbiota (infectious or not, an area that has not yet been examined in-depth), and secrete discrete, probably best-fitted, assortments of “products,” likely coming into “profiles” of BRMs (34, 52–55). This has been well-studied in the case of innate immunity to bacterial products (and live bacteria as well), using the TLR1, TLR2, TLR3, TLR4, TLR6, TLR7, and TLR9 pattern recognition receptors (PRRs) (33, 35, 56–65): platelets can sense distinct natures of danger and secrete different patterns of BRMs accordingly. For instance, LPS, targeting TLR4, was shown to stimulate a distinct intracellular signaling pathway, and elicit the secretion of distinct profiles of BRMs (54, 55). Therefore, platelets are “intelligent” in that they effectively sense the nature of a given danger and respond accordingly.
6. It has been reported recently that platelets have novel functions in vascular permeability. Certain among these have been revealed in pathology; hence, platelets and their PMPs occasionally infiltrate tissues and cause attrition and inflammation (66). Platelets have recently been identified as being largely responsible for the discerned control of vascular permeability, with extravasation of lymphocytes, activated as effectors of immunity, but not erythrocytes because they otherwise warrant the sealing of the vascular arborescence (which is the primary role of platelets) (67). Platelets have also been reported novel roles in pathology: they make leukocytes prone to release neutrophil extracellular traps (NETs) with functions in infection (e.g., sepsis) and also in cancer, favoring thrombosis in either case (68–70).
7. Platelets can bind pathogens. Platelets can sense infectious pathogens and in certain occasion bind to them, sometimes tightly, either directly or indirectly as immune complexes with antibodies or complement factors such as C5a. *In vitro*, C5b–9 is capable of inducing P-selectin expression on platelets, and both C5a and C5b–9 induce surface expression of P-selectin on endothelial cells (71–74).
8. For reasons yet unclear, some infectious pathogens can enter platelets and at least reside in them. The issue of platelet “infection” is largely unstudied, as well as the outcome of the infected platelets. The relationship between platelets and infectious pathogens has been described in recent reviews (29, 30, 35, 75–81).
9. Platelets have one of the shortest lifespan among all human cells (only certain epithelial cells are comparable to them, if one excepts the granulocytes, which dye shortly from functioning as phagocytes): this property has probably contributed to deny the “cellularity” of platelets. Platelets die as a consequence of different causes/mechanisms. Leytin (82) clearly described all events of platelet death by apoptosis, but Jackson and Schoenwaelder (83) uses the term senescent platelet death rather than apoptosis *stricto sensu*. All events of activation-associated death are necrosis, since activation-associated platelet death results in improved inflammatory receptors, release of BRMs and aggregation that causes immune reactions. Because platelets are anucleate, their apoptosis leading to cell death is intriguing (82). Two main pathways were reported, (i) intrinsic and (ii) extrinsic, that are highly regulated by intra-platelet signaling mechanisms (84). Platelet apoptosis might also play a role in hemostasis, thrombosis, and inflammatory processes (84, 85).

**Figure 1:**
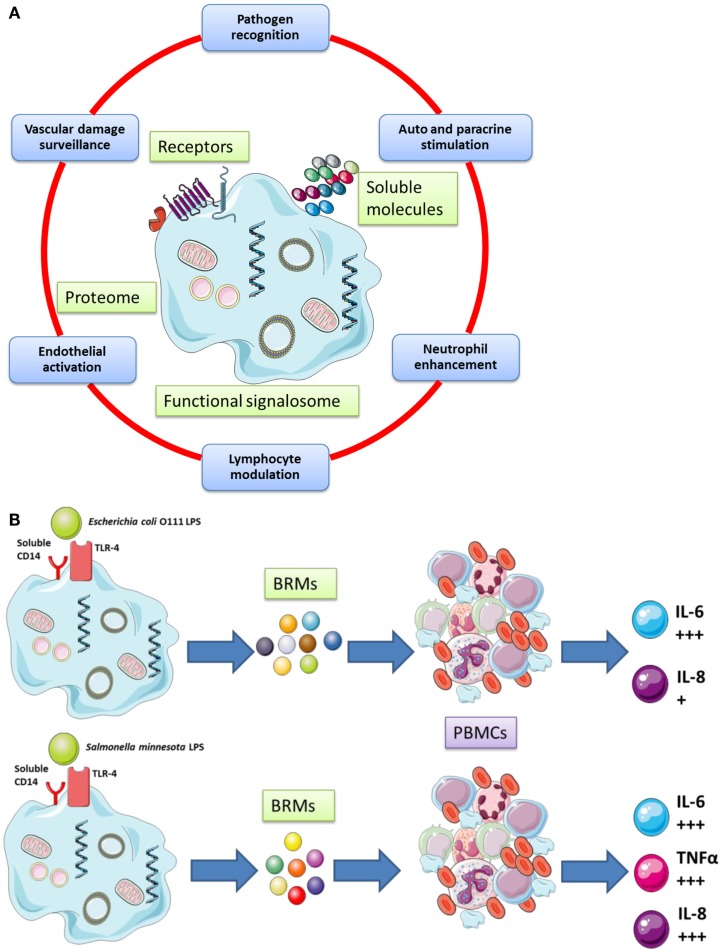
**(A)** Platelets possess important secretory functions, express internal membrane proteins, and release adhesive proteins, coagulation, and growth factors. Certain of the proteins facilitate the cross-talk of platelets with immune (e.g., leukocytes) and non-immune cells (e.g., endothelial cells). Thus, platelets play and important role in inflammatory and proliferative events and play a critical role for tissue remodeling and wound healing. **(B)** Human platelets can discriminate between various bacterial LPS isoforms via TLR4 signaling and differential cytokines secretion [adapted from Berthet et al. (54) and Hamzeh-Cognasse et al. (87)].

The cellular functions of platelets are cartooned in Figure 1 (Figure 1A gives a broad picture of the cellular functions of platelets, while Figure 1B details one selected platelet activation pathway from the sensing of a danger signal on the surface to the phosphorylation of NFκB (54) [NF-κB is a protein complex that usually controls transcription of DNA in Eukaryotes. NF-κB is found in almost all cell types involved in cellular responses to an extremely large variety of stimuli; it has NF-κB a key role in regulating the immune response to infection and – in turn – incorrect regulation of NF-κB has been linked to cancer, inflammatory, and autoimmune diseases, septic shock, viral infection, and improper immune development; it is also implicated in plasticity and cell survival (86)].

## Platelets as Immune Cells

What are “immune cells”? There is neither clear nor definite definition of an immune cell. A recent NIAID document states that: “*The immune system stockpiles a huge arsenal of cells, not only lymphocytes but also cell-devouring phagocytes and their relatives. Some immune cells take on all intruders, whereas others are trained on highly specific targets. To work effectively, most immune cells need the cooperation of their comrades. Sometimes immune cells communicate by direct physical contact, and sometimes they communicate releasing chemical messengers* […] ”^1^. According to that, platelets would be acknowledged as immune cells, but this document next stipulates that: “*All immune cells begin as immature stem cells in the bone marrow. They respond to different cytokines and other chemical signals to grow into specific immune cell types* […]”^1^; this addition would thus next deny the attribute of “immune” cell to platelets, as they do not transform themselves. Well considered, this definition does not, either, take into consideration cells and organs that are now known as being essential to optimal immune functioning, such as the microbiota for example. One may this consider that besides to key cellular actors (“Stars”) of immunity such as the lymphocytes, the phagocytes and the Ag presenting cells, there are “Supporting – though essential – roles” on stage, that are cells, which participate to immunity (such as endothelial cells, epithelial cells, and platelets).

The issue of platelets as “immune” cells has thus been not only endorsed but also extensively covered recently in a number of excellent review articles (33, 48, 88–90); therefore, we present only a brief overview and select three representative issues to address the question of platelet cellularity.

1. Platelets are innate immune sensors. As has been already presented, platelets display on their surface, and up-regulate upon stimulation, PRRs: hallmarks of innate immune functioning, beginning with the sensing of danger (28, 33, 35, 54). This property allows platelets to deal with infectious pathogens, with different outcomes depending on the nature of the invader (76, 78, 79, 81, 91–103). Platelets’ relationships with germs from the microbiota at the mucosal surfaces are suspected, but not yet deciphered.
2. Doing so, the platelets initiate inflammation. We, in fact, believe platelets are definitely inflammatory cells that exert their principal role in the physiology of vessel endothelium by detecting (sensing) dangers (vascular insults and attritions) and by fixing damage on a permanent basis. This physiological intervention and repair are no less than a healing process, which itself relates to physiological inflammation. To assist this physiological inflammation, platelets produce assortments of repair tools, such as clotting factors, cytokines, and other BRMs, growth factors, and angiogenic factors (23, 35, 75, 104–108). Apart from taht, platelets can surpass their physiological role and participate in pathological inflammation, as with cardiovascular disease, severe infection and sepsis, and arthritis (18, 23, 29, 31, 32, 36, 75, 105, 106, 109–117). Platelets, when transfused as PCs, exert their physiological and repairing role, but in 2–3% of cases (118), the physiological barrier is overcome and they release significant amounts of pro-inflammatory and directly inflammatory factors from the α and δ granules, and membrane-bound as well as solubilized or cleaved molecules. They also secrete in certain cases pro-allergenic factors (from the δ granules).
3. Platelets assist innate immunity and affect adaptive immune cells. The very first interactive role of platelets with other blood cells was, again, discovered nearly 140 years ago, with the interplay between platelets and leukocytes in thrombosis. Platelets also have extensive, though complex, interplay with leukocytes and especially polymorphonuclear cells (granulocytes) in increasing, among other things, NETs (95, 119–123). Platelets also activate monocytes and macrophages (109, 115, 124–126), T cells (127–129), B cells (28, 130–135), NK cells (136–138), and dendritic cells (DCs) (87, 93, 139–144). In turn, platelets can be activated by monocytes, T cells, B cells, and DCs (87, 89, 132, 145). This mutual interaction is not anecdotic since, for example, activated platelets can alter the isotype (Ig class) switch program of differentiated B cells (132). Moreover, platelets that harbor numerous copies of HLA class I molecules (~100,000 copies per cell) have been proposed as antigen presenting cells (this discovery, however, awaits firmer confirmation) (146). It has otherwise been suggested that more MHC-I is absorbed from the plasma by the platelets, than is derived from the platelet itself (33). Chapman et al. indicated that platelets always express significant amounts of MHC-I, but that this expression significantly increases during infection (146). Of note, those HLA class I molecules, along with variant moieties harbored by the GP molecules that mediate platelet adhesion and aggregation, termed human platelet antigens or HPA, display polymorphisms that render one’s platelets possibly immunizing when transfused into a recipient’s body (or from the fetus to the mother) (147).

In sum, platelets are not only innate and inflammatory cells themselves, but they can also assist, depending on the circumstances, adaptive immunity. They do not only assist immunity as has long been thought, but are immune cells.

## Concluding Remarks

There is no longer doubt that platelets are cells. They are also “intelligent” in that they are capable of discriminating between several types of danger and of adjusting a secretory response. This response is in general, physiologically, only that which is needed to face the danger or to fix a limited insult. This is indeed a platelet’s usual function, and what they do when they repair daily the vascular endothelium that they patrol is detect cracks and erosions, and prevent breaches and leakage (bleeding). However, the nature or extent of secretory responses by activated platelets occasionally surpasses physiological conditions and becomes pathogenic. This suggests susceptibility factors in individuals or in transfused patients, along with favorable conditions linked to causal diseases and/or therapeutics. This last issue is now regarded with renewed interest because platelet actions can be easily manipulated by drugs. If correctly readdressed, platelet activation can be turned from deleterious to beneficial to the patient in a range of different infectious and inflammatory situations such as cardiovascular disease, serious infections, autoimmune disorders, autoinflammatory diseases, and cancer.

## Conflict of Interest Statement

The authors declare that the research was conducted in the absence of any commercial or financial relationships that could be construed as a potential conflict of interest.

## Footnotes

^1^http://www.niaid.nih.gov/topics/immunesystem/Pages/immuneCells.aspx
